# Study on the preparation and properties of Sn–0.7Cu–*x*Bi alloy

**DOI:** 10.1039/d3ra06742d

**Published:** 2023-12-04

**Authors:** Zhou Shenggang, Zhang Yi, Duan Jihao, Yue Anyu, Cao Yong

**Affiliations:** a College of Materials Science and Engineering, Kunming University of Science and Technology 121 Street, Wenchang Road 68 Kunming 650093 China caoyong@kmust.edu.cn

## Abstract

This study investigated the impact of different bismuth (Bi) contents on the mechanical properties, melting point, and corrosion resistance of tin–copper (Sn–Cu) series alloys (Sn–0.7Cu). Furthermore, Sn–0.7Cu–*x*Bi alloys with different Bi contents (*x* = 0, 3, 6, 9, 12, 15 wt%) were prepared through a traditional casting process. The composition and microstructure of the alloy were characterized *via* X-ray diffraction (XRD) and Scanning electron microscopy (SEM). The impact of Bi on the mechanical properties, melting point, and corrosion resistance of Sn–0.7Cu alloy was analyzed, reaching a peak at 12 wt% Bi. Furthermore, beyond this concentration, the mechanical properties of the alloy exhibited a decline. The corrosion resistance of Sn–0.7Cu–*x*Bi alloys increased with increasing Bi content. However, when the Bi content was >12 wt%, owing to the aggregation of Bi in the alloy, the corrosion resistance of the alloy decreased.

## Abstract 2

The addition of Bi significantly enhanced the corrosion resistance of Sn–0.7Cu alloy; a Sn–0.7Cu–12Bi alloy exhibited superior corrosion resistance.

## Graphical abstract

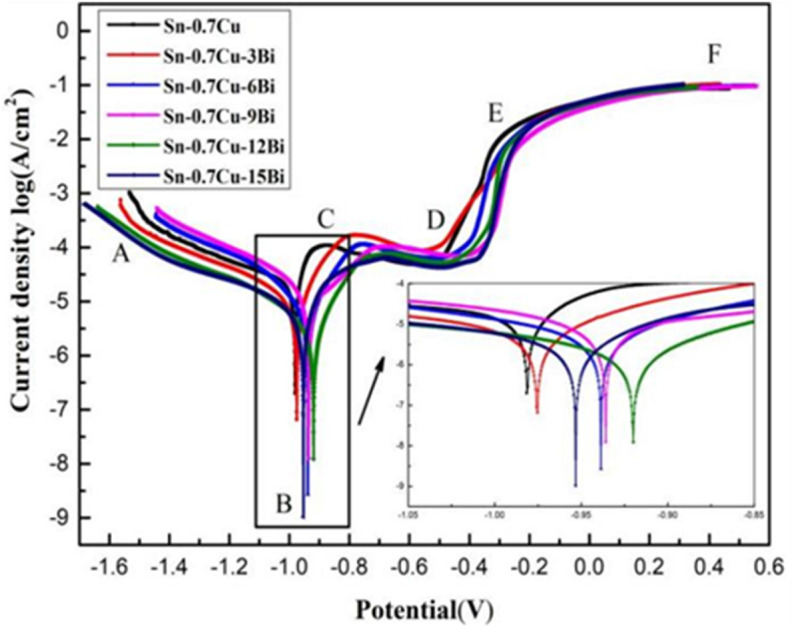

## 1. Introduction

The development and application of solder alloys have consistently captured the attention of researchers. The properties of an alloy significantly influence its utility and lifespan. In traditional solder alloys, the widespread use of tin–lead (Sn–Pb) alloy in electronic, mechanical, construction, and various other fields is attributable to the excellent performance of Pb in solder. However, the chemical toxicity of Pb limits its application, making the exploration and development of Pb-free solder a prevailing trend.^1^

To date, extensive research has been actively conducted on Pb-free alloys, particularly focusing on the corrosion resistance, mechanical properties, and physical characteristics of binary, ternary, and multicomponent Pb-free solder alloys like Sn–Cu, Sn–Ag, and Sn–Zn.^2–7^ Among these, the Sn–Cu series Pb-free alloy emerges as a promising material to replace Pb-based alloys due to its lower cost, reduced melting temperature, enhanced electrical conductivity, and ductility compared with the Sn–Ag and Sn–Zn systems.^8–10^ Nevertheless, an excess of Cu content in Sn–Cu series Pb-free alloys leads to the formation of coarse crystals, adversely affecting wettability and mechanical strength. In subsequent studies, the Sn-0.7Cu alloy, characterized by low-Cu content, lower cost, and excellent thermal fatigue properties, has garnered widespread attention.^11,12^ However, this binary alloy exhibits drawbacks such as poor wetting and mechanical properties, necessitating improvement through the addition of trace elements such as bismuth (Bi), nickel (Ni), indium (In), and others.^13^ For example, Yang *et al.* elucidated the impact of rare earth elements such as cerium (Ce), Ag, and Bi on the wetting and mechanical properties of Sn–Cu–Ni Pb-free solder.^14^ The addition of these elements has demonstrated a certain degree of improvement in alloy properties, indicating the positive effect of elements such as Bi on the mechanical properties.

Nevertheless, despite advancements in alloy composition, the progress of corrosion poses potential risks to personal safety and environmental health.^15^ Consequently, solder materials must exhibit significant corrosion resistance to ensure high connection reliability. At present, research on the corrosion resistance of solder is ongoing.^16^ For example, Gao *et al.* investigated the corrosion behavior of Sn–0.75Cu solder in 3.5 wt% NaCl solution, revealing the formation of pits on the solder surface.^15^ Similarly, Dheeraj *et al.* examined the corrosion phenomenon of Sn–9Zn–*x*Cu solder alloy in 3.5 wt% NaCl solution, discussing the possible causes of Zn corrosion products and proposing accurate mechanisms.^17^ Studies have shown that the addition of Bi to Sn–0.7Cu alloy affects the electrochemical performance of the alloy to a certain extent, with limited research in this area.

Notably, no systematic study has been conducted on the influence of varying Bi concentrations (0, 3, 6, 9, 12, and 15 wt%) in Sn–0.7Cu on the mechanical properties, corrosion resistance, and other comprehensive properties of Sn–0.7Cu–*x*Bi alloys. Therefore, this study aimed to explore the effect of adding Bi on the structure, melting characteristics, mechanical properties, and corrosion resistance of Sn–0.7Cu–*x*Bi alloy. Additionally, the corrosion state and mechanism in a 3.5% NaCl solution were analyzed.

## 2. Materials and methods

### 2.1. Alloy preparation

Six types of Sn–0.7Cu–*x*Bi alloys with different Bi content were synthesized using Sn, Bi, and Cu (purity > 99.99%). The alloy compositions are shown in Table 1. In this experiment, the alloy was prepared *via* the traditional casting process. Initially, the raw materials were immersed in 1 mol L^−1^ hydrochloric acid for 5–10 min to eliminate oxide films. Afterward, the materials were thoroughly rinsed and dried for subsequent use. Subsequently, the dried Sn–Cu alloy was placed into a porcelain crucible and then heated to 350 °C for melting in a well-type resistance furnace under protective gas.^18^ Bi was then introduced into the molten alloy, stirred every 15 min, and a specific amount of covering agent (KCl + LiCl) was evenly sprinkled on the surface for coverage. The mixture was kept warm for 1 hour under argon protection.^19^ Finally, the metal mixture, cooled to 300 °C, was poured into a graphite mold preheated at 150 °C and allowed to cool at room temperature to obtain the desired shape.

**Table 1:** The measured and calculated values of the density of the prepared alloys

Alloy	Code	Density (g cm^−3^)	
		Measured	Calculated
Sn : Cu = 99.3 : 0.7	S1	7.108	7.290
Sn : Cu : Bi = 96.3 : 0.7 : 3	S2	7.278	7.346
Sn : Cu : Bi = 93.3 : 0.7 : 6	S3	7.395	7.404
Sn : Cu : Bi = 90.3 : 0.7 : 9	S4	7.448	7.463
Sn : Cu : Bi = 87.3 : 0.7 : 12	S5	7.494	7.521
Sn : Cu : Bi = 84.3 : 0.7 : 15	S6	7.569	7.582

### 2.2. Microstructural morphology and mechanical properties test

To observe the microstructure of the composite alloy, the melted alloy was cut into small squares (10 × 10 × 3 mm), polished successively with 600 #, 1000 #, 1500 #, 2000 #, and 2500 # water abrasive paper, and then polished with alumina polishing powder (0.5 μ particle size) until the alloy surface was so smooth as possible. After polishing, the alloy was subjected to ultrasonic cleaning for 30 min. Microstructure and morphology were observed *via* Scanning electron microscopy (SEM, Nova Nano), and element content and distribution were analyzed *via* Energy-dispersive X-ray energy spectrometry (EDS).

Then, an X-ray diffractometer (XRD-7000) was used for phase analysis of the alloy to obtain its phase composition. Cu-Kα and 2*θ*/*θ* scanning modes were employed to characterize the deposition patterns within the range of 20–80°, with a scanning speed of 6° min^−1^. The measured XRD data were imported into Jade software and compared with standard cards to identify the phase composition of the alloy and a comparative analysis was conducted with the EDS results.

The Vickers hardness tester was employed to test the hardness value of the alloy under a specified load (*F* = 9.8 N). Additionally, a universal testing machine (SLFL-100KN) was utilized to conduct tensile tests at room temperature, generating stress–strain curves. The morphology of fracture surfaces was characterized using SEM.

*Via* differential scanning calorimetry (TGA/DSC/1600LF) in the temperature range of 25–300 °C under atmospheric nitrogen conditions with a flow rate of 20 ml min^−1^, and a heating rate is 10 °C min^−1^.

### 2.3. Electrochemical corrosion test

Electrochemical testing (CS2350H) of Sn–0.7Cu–*x*Bi alloy was conducted using a classic three-electrode system. A 3.5 wt% NaCl solution served as the corrosion test solution. The previously polished Sn–0.7Cu–*x*Bi alloy, connected using wires and sealed with epoxy resin, was used as the working electrode. A platinum electrode with an equivalent test area served as the auxiliary electrode, and a saturated calomel electrode was the reference electrode, forming a three-electrode system. Furthermore, before electrochemical impedance spectroscopy (EIS) measurement, an open circuit potential (OCP) test of 1800 s was conducted to confirm the stability of the working surface electrode. The polarization scanning range for this test was –1 V–1 V, with a scanning rate of 0.5 mV s^−1^,^15,17^ to obtain the potentiodynamic polarization curve of the alloy sample. Similarly, using CS Studio5 software, the polarization curve was fitted to obtain the self-correction potential (*E*_corr_) and self-correction current density (*j*_corr_). An alternating current impedance test was conducted within the range of 0.01–100 kHz. After the test, appropriate equivalent circuits were selected using ZismDemo3.30d software for fitting and analyzing EIS data. Thus, electrochemical testing, SEM, EDS, and XRD were used to analyze the electrochemical corrosion morphology, elemental distribution, and phase composition of the alloy.

## 3. Results and discussion

### 3.1. Microstructure and EDS analysis

**Fig. 1:**
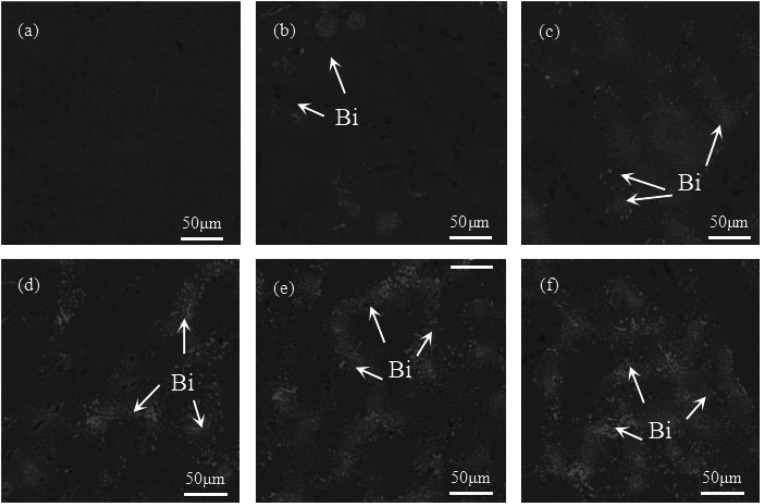
Microstructure and morphology of Sn–0.7Cu–*x*Bi alloy: (a) Sn–0.7Cu; (b) Sn–0.7Cu–3Bi; (c) Sn–0.7Cu–6Bi; (d) Sn–0.7Cu–9Bi; (e) Sn–0.7Cu–12Bi; (f) Sn–0.7Cu–15Bi.

Fig. 1 illustrates the microstructure and morphology of Sn–0.7Cu–*x*Bi alloys with different Bi contents. Fig. 1(a) depicts the microstructure and morphology of Sn–0.7Cu alloy, where scattered dark black tissues were distributed on the gray Sn matrix. These dark black tissues corresponded to the intermetallic compound Cu_6_Sn_5_,^18^ as inferred from the binary phase diagram of Sn–Cu. Subsequently, figures (Fig. 1(b–f)) revealed significant alterations in the microstructure of the Sn–0.7Cu–*x*Bi alloys due to the incorporation of Bi. The Bi-containing alloys exhibited a distinct Sn–Bi structure morphology, comprising white Bi phases and gray Sn solid solution with a lamellar distribution.

In a comparative analysis of microstructures in Fig. 1(b), (d), and (f), it was observed that when the Bi content in the alloy was 3 wt%, there was only a small amount of fine Bi phase on the surface of the Sn matrix. At this time, Bi strengthened the second phase of the alloy and significantly improved the properties of the alloy. At 9 wt% Bi content, a uniform distribution of fine Bi phase with a fine needle-like pattern structure was observed, leading to further enhancement of alloy properties through dispersion strengthening. However, as the Bi content increases to 15 wt%, the limited solubility of Bi in Sn resulted in the precipitation of a large amount of Bi phase on the Sn base surface. This led to the coarsening and segregation of Bi, ultimately diminishing the alloy properties.

**Fig. 2:**
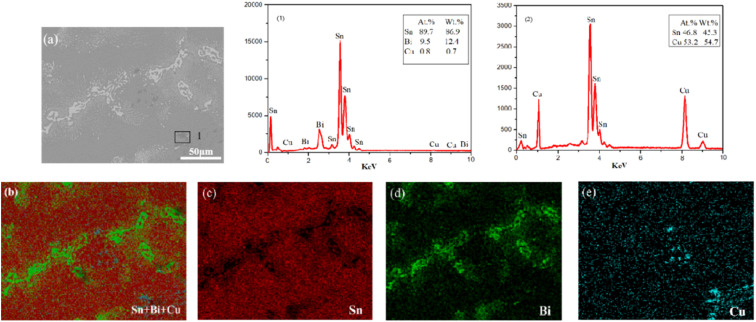
EDS elemental mapping analysis of Sn–0.7Cu–12Bi alloy: (b) Sn + Bi + Cu; (c) Sn; (d) Bi; (e) Cu.

To further analyze the alloy element composition, an energy spectrum analysis was conducted on Sn–0.7Cu–12Bi alloy with 12 wt% Bi. Fig. 2 (1) revealed that the mass fraction of Sn in the alloy was 86.9 wt%, the mass fraction of Bi was 12.4 wt%, and the mass fraction of Cu was 0.7 wt%. This confirmed the uniform distribution of alloy elements during the refining process. Additionally, the energy spectrum analysis of Fig. 2(a) indicated that Sn was primarily distributed in the Sn-rich phase, while Cu was predominantly present in Cu_6_Sn_5_ intermetallic compounds.^14^ Furthermore, the addition of 12 wt% Bi resulted in the accumulation of pure Bi precipitates on the Sn phase surface, evenly distributed throughout the microstructure.

### 3.2. XRD analysis

**Fig. 3:**
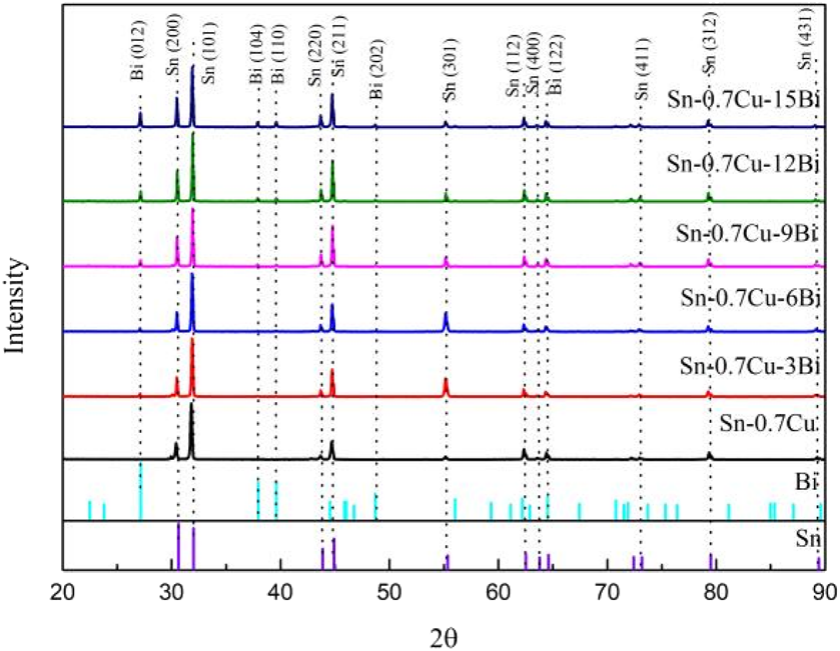
XRD diffraction patterns of Sn–0.7Cu–*x*Bi alloy.

To determine the element composition of Sn–0.7Cu–*x*Bi alloy, XRD analysis, and EDS analysis were conducted on alloys with different Bi contents. Fig. 3 shows the XRD results of alloys with different Bi contents. In Fig. 3, only the peak of the Sn phase was detected for Sn–0.7Cu, while with the addition of Bi, diffraction peaks of Bi phases became wider and higher, indicating the presence of Bi-rich phases. Alam *et al.* found that the addition of Bi to Sn–0.7Cu formed a Bi phase in Sn, and no new compounds were formed.^18^ The XRD diffraction patterns revealed that owing to the low amount of Cu in the alloy, there was no corresponding diffraction peak of the Cu. The XRD results confirmed the existence of Sn-rich and Bi-rich phases in Sn–0.7Cu–*x*Bi alloy, consistent with phase diagram predictions for Sn–Bi–Cu alloy. The weak diffraction peaks of the formed Cu_6_Sn_5_ phase were not evident in the XRD patterns.^14^

### 3.3. Mechanical properties

#### 3.3.1. Tensile mechanical properties test

**Fig. 4:**
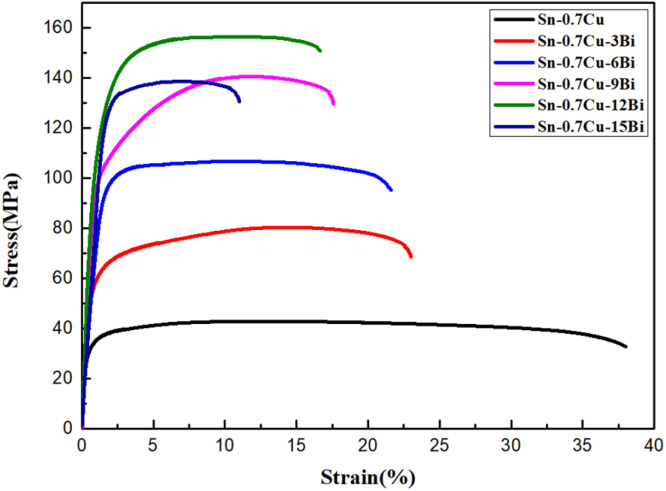
Tensile stress–strain curve of Sn–0.7Cu–*x*Bi alloy at room temperature.

Fig. 4 shows the tensile stress–strain curve of Sn–0.7Cu–*x*Bi alloy at room temperature. From Fig. 4, it was observed that the alloy exhibited only elastic deformation and yield deformation stages, without a necking stage. After data processing, the strength and fracture elongation of the composite alloy in Table 2 were obtained. From the table, it was evident that the tensile properties of the alloy were significantly affected by Bi. The yield strength and tensile strength of the alloy revealed a trend of initially increasing and then decreasing with the increase of Bi content, while its fracture elongation gradually decreased, reaching the lowest value of 11.74% when the Bi content reached 15 wt%. The yield strength and tensile strength of Sn–0.7Cu alloy were the lowest, with only 29.2 MPa and 42.97 MPa, while its fracture elongation reached the maximum value of 37.61%. The yield strength and tensile strength of the alloy reached the maximum when the Bi content was 12 wt%, which was 100 MPa and 156.24 MPa, and the elongation at break was 17.18%. This phenomenon was attributable to the fact that when the Bi content was <12 wt%, the addition of Bi to the composite alloy played the role of grain refinement and dispersion strengthening. Therefore, the yield strength and tensile strength of the alloy increased gradually. However, the addition of excessive Bi led to a large accumulation on the Sn base, and the Supersaturation Bi segregated and coarsened in the alloy. Similarly, as a hard brittle phase, Bi increased the strength and reduced the elongation of the alloy, leading to more crack initiation areas during the tensile test, thereby reducing the strength of the alloy. Additionally, considering both the strength and fracture elongation at the break of the alloy, when the Bi content was 12 wt%, the comprehensive mechanical properties of Sn–0.7Cu–*x*Bi alloy were excellent.

**Table 2:** Tensile property parameters of Sn–0.7Cu–*x*Bi alloy at room temperature

Sample	Yield strength (MPa)	Tensile strength (MPa)	Elongation (%)
Sn–0.7Cu	29.2	42.97	37.61
Sn–0.7Cu–3Bi	48.9	80.39	23.44
Sn–0.7Cu–6Bi	91.8	105.41	22.13
Sn–0.7Cu–9Bi	95.9	140.57	17.58
Sn–0.7Cu–12Bi	100	156.24	17.18
Sn–0.7Cu–15Bi	98.4	137.51	11.74

#### 3.3.2. Stretch fracture morphology test

**Fig. 5:**
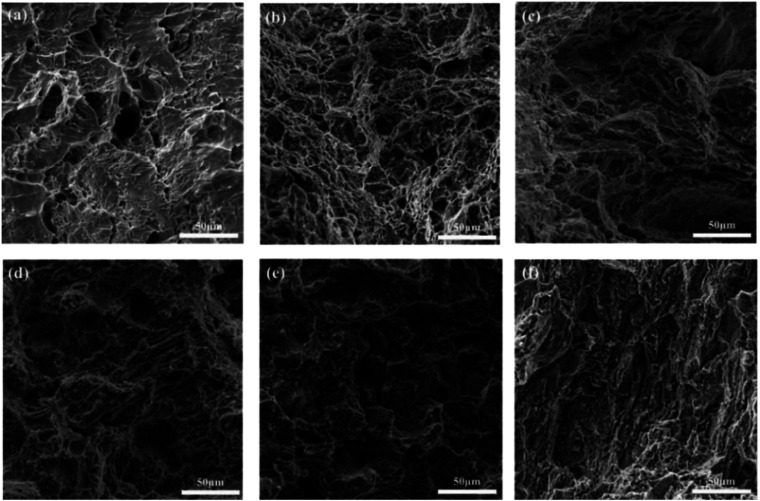
Tensile fracture topography of Sn–0.7Cu–*x*Bi alloy at room temperature: (a) Sn–0.7Cu; (b) Sn–0.7Cu–3Bi; (c) Sn–0.7Cu–6Bi; (d) Sn–0.7Cu–9Bi; (e) Sn–0.7Cu–12Bi; (f) Sn–0.7Cu–15Bi.

To understand the effect of different Bi contents on the fracture behavior of Sn–0.7Cu–*x*Bi alloy, confirming its fracture mode and fracture mechanism, the fracture morphology of Sn–0.7Cu–*x*Bi alloy after tension at room temperature was analyzed *via* SEM. The micro-scanning image was obtained as shown in Fig. 5. It was observed that the addition of Bi had a significant influence on the fracture morphology of Sn–0.7Cu–*x*Bi alloy. From Fig. 5(a–c), it was observed that there were dimple bands gathered on the fracture surface, with large dimples intermingled with a large number of small dimples. The tearing patterns were clear, with obvious secondary cracks, small step-like cleavage surfaces, and longer tear edges. The ductile fracture of the alloy was always accompanied by a significant level of plastic deformation, which was regarded as the flow of metal in the tensile dimple. In the uniaxial tensile test, a large number of small holes were produced due to the three-dimensional stress state. Given the continuous application of load, these micropores were connected, resulting in a central crack that led to the fracture of the alloy. Therefore, micropores and dimples were regarded as signs of ductile fracture of the alloy when the Bi content was low. However, as shown in Fig. 5(f), when the Bi content increased to 15 wt%, some cracks, and flat cleavage areas gradually appeared on the port surface of the alloy, and the fracture surface revealed a pyramid-shaped shape with clear edges and corners, indicating a polyhedral shape. The appearance of this fracture morphology indicated that the fracture mode of the alloy shifted from ductile fracture to brittle fracture as the content of Bi in the alloy increased continuously. This phenomenon was attributable to the accumulation of Bi as a hard and brittle phase at the fracture site of the alloy, and the brittle-rich Bi phase experienced intergranular fracture, leading to brittle fracture. This phenomenon corresponded to the stress–strain curve of the alloy in tension, where the fracture elongation decreased while the strength increased with the addition of Bi.

#### 3.3.3. Hardness test

**Fig. 6:**
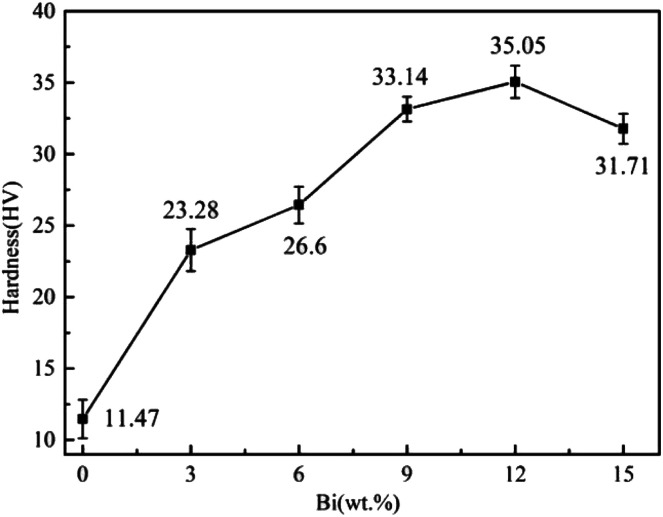
Vickers hardness of Sn–0.7Cu–*x*Bi alloy.

The Vickers hardness of the Sn–0.7Cu–*x*Bi alloy with different Bi contents is shown in Fig. 6. It was evident that as the Bi composition in the alloy increased, the hardness of the Sn–0.7Cu–*x*Bi alloy exhibited a progressively upward trend. Specifically, it increased from 11.47 HV for pure Sn–0.7Cu alloy to 35.05 HV for Sn–0.7Cu–12Bi alloy. However, when the Bi content increased to 15 wt%, the hardness of the Sn–0.7Cu–15Bi alloy decreased slightly to 31.71 HV. It was observed that the hardness of the alloy significantly improved by adding an appropriate amount of Bi. The relationship between the strength and hardness of the alloy corresponded to the previously discussed trend in the tensile properties of the alloys. The underlying reason for this trend was that when the Bi content in the alloy was <12 wt%, a small amount of Bi was uniformly distributed in the Sn base as tiny, dispersed particles, effectively impeding the movement of dislocations and thereby continuously increasing the hardness of the alloy. However, as the Bi content increased, the accumulation and clustering of Bi as hard and brittle phases occurred, leading to a gradual reduction in resistance to dislocation movement. Therefore, the hardness of the Sn–0.7Cu–15Bi alloy was lower than that of the Sn–0.7Cu–12Bi alloy. Given that the hardness of Bi was higher than that of the Sn matrix, the precipitated Bi element played the role of second-phase strengthening in the alloy. Therefore, the hardness of alloys with added Bi was higher than that of the pure Sn–0.7Cu alloy.

### 3.4. Melting properties test

**Fig. 7:**
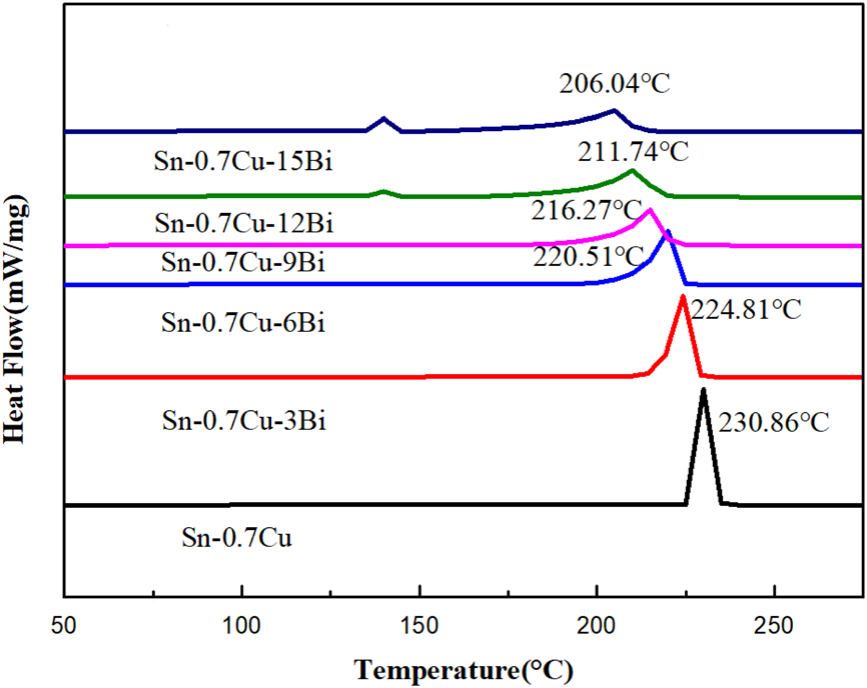
DSC curve of Sn–0.7Cu–*x*Bi alloy.

To study the effect of Bi content on the melting properties of the Sn–0.7Cu alloy, the melting properties of all six types of alloys were measured using DSC. Fig. 7 depicts the DSC curve of Sn–0.7Cu–*x*Bi alloy. Some melting performance parameters and calculated melting range were obtained from the DSC curve (Table 3). In Fig. 7, it was evident that the Sn–0.7Cu–*x*Bi alloy was non-eutectic. Unlike eutectic alloys, the melting process of non-eutectic alloys occurred within a specific melting range, rather than completing the solid–liquid transformation at a single point. When the Bi content in the alloy was <6 wt%, a solitary endothermic peak at 225 °C was observed on the DSC curve. However, when the Bi content exceeded 6 wt%, a weak endothermic peak emerged at 140 °C. Additionally, the previous peak and endothermic area peak increased with increased Bi content. According to the Sn–Bi binary phase diagram, this phenomenon was attributable to the segregation of the Bi portion, forming a solid solution with the matrix Sn at 140 °C, resulting in the formation of a small number of eutectic phases.

**Table 3:** DSC curve analysis results of Sn–0.7Cu–*x*Bi alloy

Alloy	Liquidus 1 (°C)	Melting point 1 (°C)	Solidus 1 (°C)	Liquidus 2 (°C)	Melting point 2 (°C)	Solidus 2 (°C)	Melting range (°C)
Sn–0.7Cu	—	—	—	227.15	230.86	233.27	4.3
Sn–0.7Cu–3Bi	—	—	—	218.94	224.81	227.30	6.08
Sn–0.7Cu–6Bi	139.05	139.56	140.49	212.27	220.51	223.13	8.58
Sn–0.7Cu–9Bi	139.06	139.68	140.72	206.32	216.27	220.16	11.51
Sn–0.7Cu–12Bi	139.07	139.82	140.81	199.11	211.74	216.37	14.83
Sn–0.7Cu–15Bi	138.98	140.05	141.18	191.85	206.04	210.59	18.50

Furthermore, the DSC curve results indicated that the addition of Bi element had minimal effect on the small peak melting point of the alloy (Table 3). The small peak melting point of the Sn–0.7Cu–6Bi alloy was only 0.49 °C lower than that of the Sn–0.7Cu–15Bi alloy. This indicated that the melting point of the eutectic phase was relatively stable and slightly affected by the Bi content. Nevertheless, the melting point of the alloy, the melting point, solidus, and liquidus of Sn–0.7Cu alloy significantly decreased with the increase of Bi element content. The melting point decreased from 230.86 °C in the initial Sn–0.7Cu alloy to 206.04 °C in the Sn–0.7Cu–15Bi alloy, reducing 10.75%. Shen *et al.* also reported that adding Bi lowers the melting point of the Sn–Cu solder alloy.^12^ The low melting point nature of Bi, as a fusible alloy, led to the formation of a low melting point phase with Sn upon Bi addition, resulting in a continuous decrease in the melting point of the alloy. Further comparison of the melting range revealed an increase with higher Bi content.

### 3.5. Electrochemical corrosion performance test

#### 3.5.1. Open circuit potential test

**Fig. 8:**
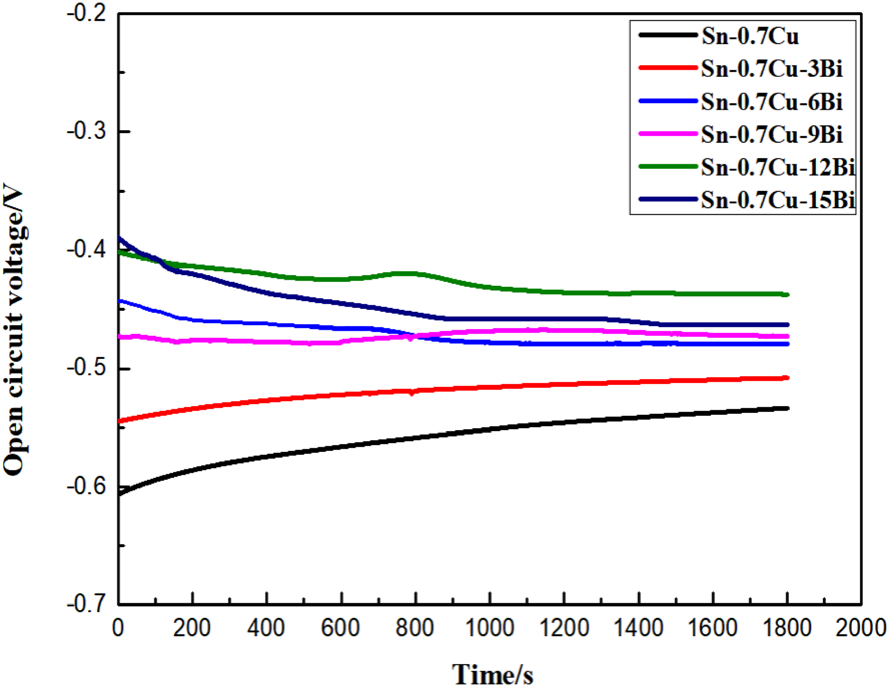
OCP test of Sn–0.7Cu–*x*Bi alloy in 3.5 wt% NaCl solution.

The OCP test results of Sn–0.7Cu–*x*Bi alloy are shown in Fig. 8. From the figure, it was observed that the OCP in the NaCl solution was stabilized, and a gradual increase was also observed with the addition of the Bi element. The OCP reached its maximum value at a Bi content of 12 wt%. Although the OCP decreased was <12 wt% Bi, it remained significantly higher than that of pure Sn–0.7Cu alloy. Therefore, the addition of Bi significantly improved the corrosion resistance of Sn–0.7Cu alloy. From a corrosion thermodynamics perspective, the magnitude of the OCP reflected the electrochemical corrosion trend of the material under unloaded conditions. A more positive OCP value indicated a smaller corrosion tendency in the corrosion solution. Consequently, it was concluded that Sn–0.7Cu–12Bi alloy was the most stable in a 3.5 wt% NaCl solution. However, OCP alone cannot fully represent the corrosion resistance of the sample. Therefore, the corrosion resistance of Sn–0.7Cu–*x*Bi alloy in a 3.5 wt% NaCl solution was further elucidated through subsequent analysis of polarization curves and EIS.

#### 3.5.2. Potentiodynamic polarization test

**Fig. 9:**
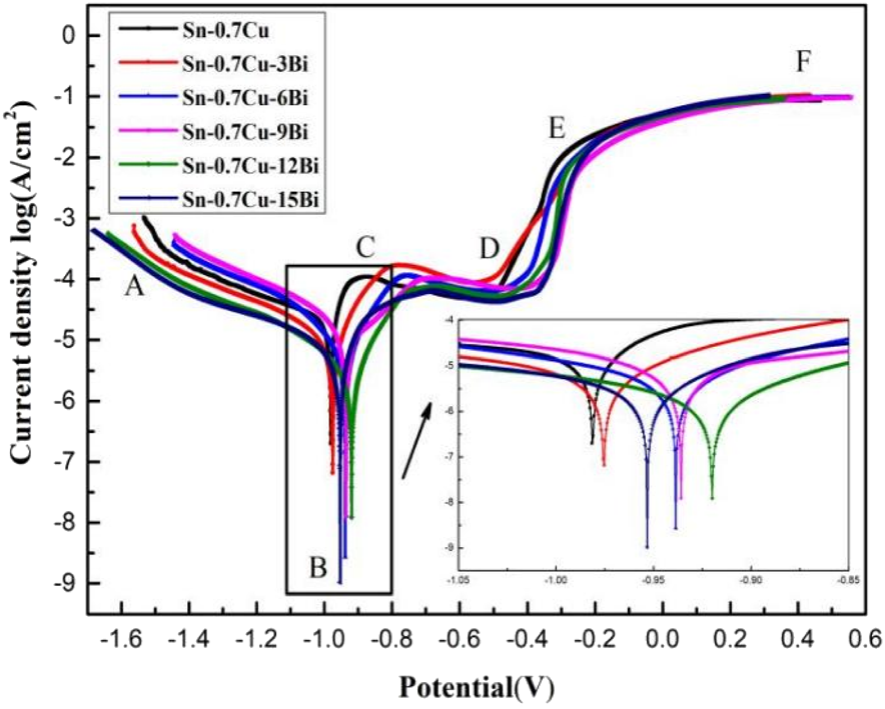
Potentiodynamic polarization curve of Sn–0.7Cu–*x*Bi alloy in 3.5 wt% NaCl solution.

To determine the optimal amount of Bi in Sn–0.7Cu–*x*Bi alloy, potentiodynamic polarization curve testing was conducted on the corrosion resistance of the alloy. Fig. 9 shows the potentiodynamic polarization curve of Sn–0.7Cu–*x*Bi alloy in a 3.5 wt% NaCl solution. It was evident that the change of self-corrosion potential of the alloy was consistent with that of the OCP. The addition of Bi shifted the potential of the alloy in a positive direction. The polarization curve consisted of an anode branch and a cathode branch. Points A to B represent polarized cathode branches, owing to the reduction of dissolved oxygen, as reported by Jaffery *et al.*^20,21^ The anode reaction stage involves regions BC, CD, DE, and EF, respectively. Generally, the diffusion process of Sn activity started from point B, and the corrosion current density increased until the corrosion product reached the critical value on the surface (point C). Then, these corrosion products covered the surface to prevent further corrosion of the alloy, and a plateau area (CD) appeared on the curve.^22^ When the potential reached point D, the current density increased significantly, indicating the breakdown of the passive film.

Furthermore, upon fitting the Tafel curve, the corrosion parameters of Sn–0.7Cu–*x*Bi alloy were obtained (Table 4). Table 4 shows that the self-corrosion potential of Sn–0.7Cu–*x*Bi alloy reached −0.92 V when the addition of Bi element was 12 wt%. From the perspective of corrosion thermodynamics, the higher the self-corrosion potential of an alloy, the smaller its corrosion tendency, indicating that the alloy was more stable and less susceptible to corrosion in a 3.5 wt% NaCl solution. However, this alone does not truly reflect the corrosion resistance of the alloy. In terms of alloy corrosion kinetics, the self-corrosion current density reflected the degree of corrosion in the NaCl solution. A lower self-corrosion current density corresponds to a slower corrosion rate. Table 4 demonstrates that at 12 wt% Bi, the alloy exhibited the lowest self-corrosion current density, 5.132 × 10^−6^. The polarization resistance (*R*_p_) of the alloy supported this, with the higher polarization resistance observed at 12 wt% Bi, indicating superior corrosion resistance. Compared with pure Sn–0.7Cu alloy, the corrosion resistance of Sn–0.7Cu–12Bi alloy was significantly improved. In summary, the analysis indicated that adding an appropriate amount of Bi (<12 wt%) to the alloy enhanced grain refinement and dispersion strengthening, preventing corrosion erosion. This addition was beneficial to improve the corrosion resistance of the alloy. However, when the Bi content was >12 wt%, the coarsening of Bi elements intensified galvanic corrosion, selectively corroding the Sn matrix, leading to decreased corrosion resistance.

**Table 4:** Fitting parameters of polarization curve of Sn–0.7Cu–*x*Bi alloy in 3.5 wt% NaCl solution

Sample	*E*_corr_ (mV)	*j*_corr_ (A cm^−2^)	*R*_p_ (Ω cm^2^)
Sn–0.7Cu	−981.25	3.465 × 10^−5^	3.276 × 10^3^
Sn–0.7Cu–3Bi	−975.59	2.401 × 10^−5^	4.263 × 10^3^
Sn–0.7Cu–6Bi	−938.55	1.464 × 10^−5^	4.869 × 10^3^
Sn–0.7Cu–9Bi	−936.28	1.116 × 10^−5^	4.597 × 10^3^
Sn–0.7Cu–12Bi	−920.08	5.132 × 10^−6^	12.012 × 10^3^
Sn–0.7Cu–15Bi	−953.35	2.089 × 10^−5^	7.935 × 10^3^

#### 3.5.3. Electrochemical impedance test

**Fig. 10:**
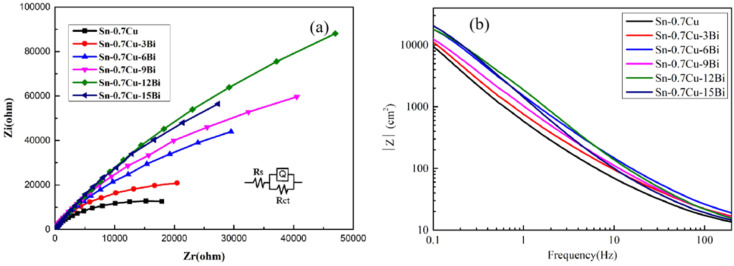
(a) EIS diagram and (b) Bode plots of Sn–0.7Cu–*x*Bi alloy in 3.5 wt% NaCl solution.

The EIS curve and Bode plots of the prepared Sn–0.7Cu–*x*Bi alloy in a 3.5 wt% NaCl solution is shown in Fig. 10. To evaluate the EIS parameters, the EIS data were fitted using ZSimDemo3.30d software, adopting the *R*_s_ (*QR*_ct_) equivalent circuit model. This equivalent circuit is shown in Fig. 10 (a), with *R*_s_ denoting solution resistance, *R*_ct_ representing charge transfer resistance, and *Q* representing the constant phase element. The fitting results are summarized in Table 5, and the fitting errors for each alloy were found to be <10^−4^, indicating strong agreement between the test results and the fitting data. Furthermore, examining the Nyquist diagram of Fig. 10(a), it was evident that the Bi content of the alloy increased, the diameter of the semicircle in the Nyquist curve gradually increased, and the semicircle diameter of the Sn–0.7Cu–12Bi alloy exhibited the largest semicircle diameter. The larger the diameter of the semicircle in the Nyquist curve, the stronger the corrosion resistance. Furthermore, the Bode diagram of |*Z*| *versus* frequency in Fig. 10(b) revealed that at low frequencies, the Sn–0.7Cu–12Bi alloy possessed the highest |*Z*| value, corresponding to the largest capacitance semicircle. These observations aligned with the results of polarization analysis, indicating that when the Bi content reached 12 wt%, the corrosion resistance of the matrix alloy was at its peak. Table 5 displays the order of *R*_ct_ values for the alloy as Sn–0.7Cu–12Bi > Sn–0.7Cu–15Bi > Sn–0.7Cu–9Bi > Sn–0.7Cu–6Bi > Sn–0.7Cu–3Bi > Sn–0.7Cu. Higher *R*_ct_ values typically indicated reduced charge transfer on the alloy surface, indicating lower corrosion susceptibility. The Sn–0.7Cu–12Bi alloy exhibited the highest *R*_ct_ value and the strongest corrosion resistance, which was consistent with the results of OCP and potentiodynamic polarization curves in the previous study.^23^ The trend in the *R*_ct_ value was consistent with the previous *R*_p_ value. The primary reason for this enhanced corrosion resistance was attributable to the grain-refining effect of the Bi in the Sn matrix and its ability to homogenize the alloy.

**Table 5:** EIS curve fitting parameters of Sn–0.7Cu–*x*Bi alloy

Sample	*R*_s_ (Ω cm^2^)	CPE	*n*	*R*_ct_ (Ω cm^2^)
Sn–0.7Cu	7.42	3.76 × 10^−5^	0.89	2.93 × 10^4^
Sn–0.7Cu–3Bi	8.69	3.44 × 10^−5^	0.91	4.65 × 10^4^
Sn–0.7Cu–6Bi	7.88	2.02 × 10^−5^	0.88	1.11 × 10^5^
Sn–0.7Cu–9Bi	7.39	1.61 × 10^−5^	0.86	1.76 × 10^5^
Sn–0.7Cu–12Bi	13.73	2.28 × 10^−5^	0.81	5.08 × 10^5^
Sn–0.7Cu–15Bi	7.19	1.34 × 10^−5^	0.82	3.67 × 10^5^

However, when the added Bi exceeded the solid solubility in Sn, *R*_ct_ tended to decrease. Excessive Bi was therefore detrimental to the corrosion resistance of Sn–0.7Cu–*x*Bi alloy. Similarly, Li *et al.* reported that an excess of Bi reduced the corrosion resistance of Sn–Cu alloy.^13^ This was attributable to the agglomerated Bi accumulation on the surface of Sn, which aggravated the pitting corrosion and reduced the corrosion resistance of the alloy.

#### 3.5.4. Characterization of corrosion product and corrosion mechanism

**Fig. 11:**
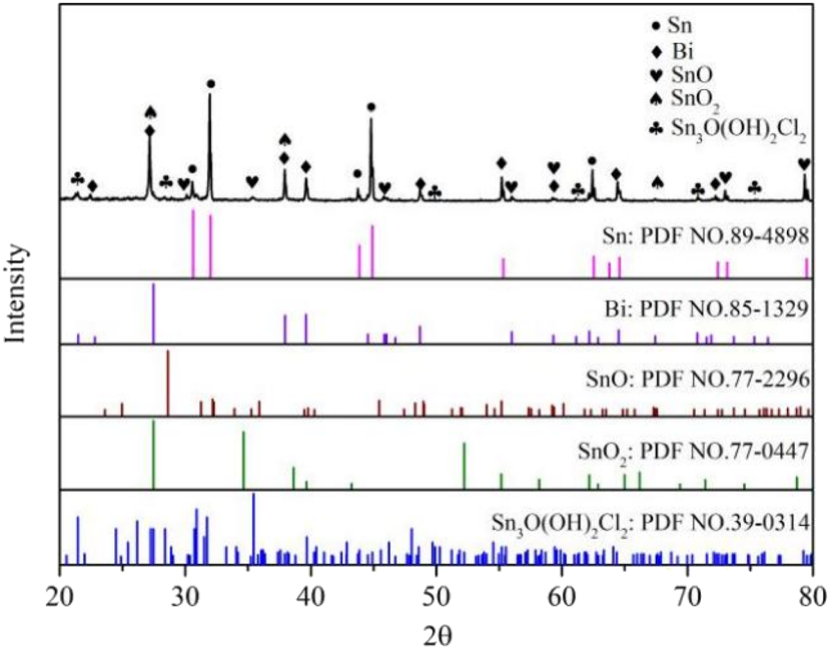
XRD diffraction pattern of Sn–0.7Cu–*x*Bi alloy after electrochemical corrosion.

To determine the corrosion products of Sn–0.7Cu–*x*Bi alloy after electrochemical corrosion, the XRD tests were conducted on the corroded alloy, and the test results are shown in Fig. 11. It was observed from the diagram that the corroded alloy exhibited three more phases of SnO, SnO_2_, and Sn_3_O (OH)_2_Cl_2_ compared with the uncorroded alloy. This phenomenon was attributable to the use of a neutral NaCl solution in the electrochemical corrosion process, where the main reaction of the cathode involved oxygen dissolution and subsequent hydrogen evolution.

1 O_2_ + 2H_2_O = 4OH^−^ + 4e^−^

2 2H_2_O = 2OH^−^ + H_2_ + 2e^−^

The anodic reaction mainly involved the dissolution of active metals. Because the content of Cu in the alloy was low, Bi as the second phase in the alloy was still pure in the single phase. Therefore, the anodic reaction was the dissolution of Sn. Thermodynamically, Sn oxide was readily formed, making Sn hydroxide dehydrate to form Sn oxide.^24–27^ The specific reaction process was as follows:

3 Sn + 2OH^−^ = Sn(OH)_2_ + 2e^−^

4 Sn(OH)_2_ = SnO + H_2_O

5 Sn(OH)_2_ + 2OH^−^ = Sn(OH)_4_ + 2e^−^

6 Sn(OH)_4_ = SnO_2_ + 2H_2_O

From Fig. 11, it was evident that the corrosion products of Sn–0.7Cu–*x*Bi alloy after electrochemical corrosion exhibited diffraction peaks of Sn_3_O (OH)_2_Cl_2_ phase in addition to SnO and SnO_2_ phases, consistent with the results findings of Jaiswal *et al.*^28^ The chemical reaction equation is as follows:^29,30^

7 3Sn + 4OH^−^ + 2Cl^−^ = Sn_3_O(OH)_2_Cl_2_ + H_2_O + 6e^−^

Therefore, the XRD analysis indicated that the corrosion products of the composite alloy mainly consisted of three compounds: SnO, SnO_2_, and Sn_3_O(OH)_2_Cl_2_.

**Fig. 12:**
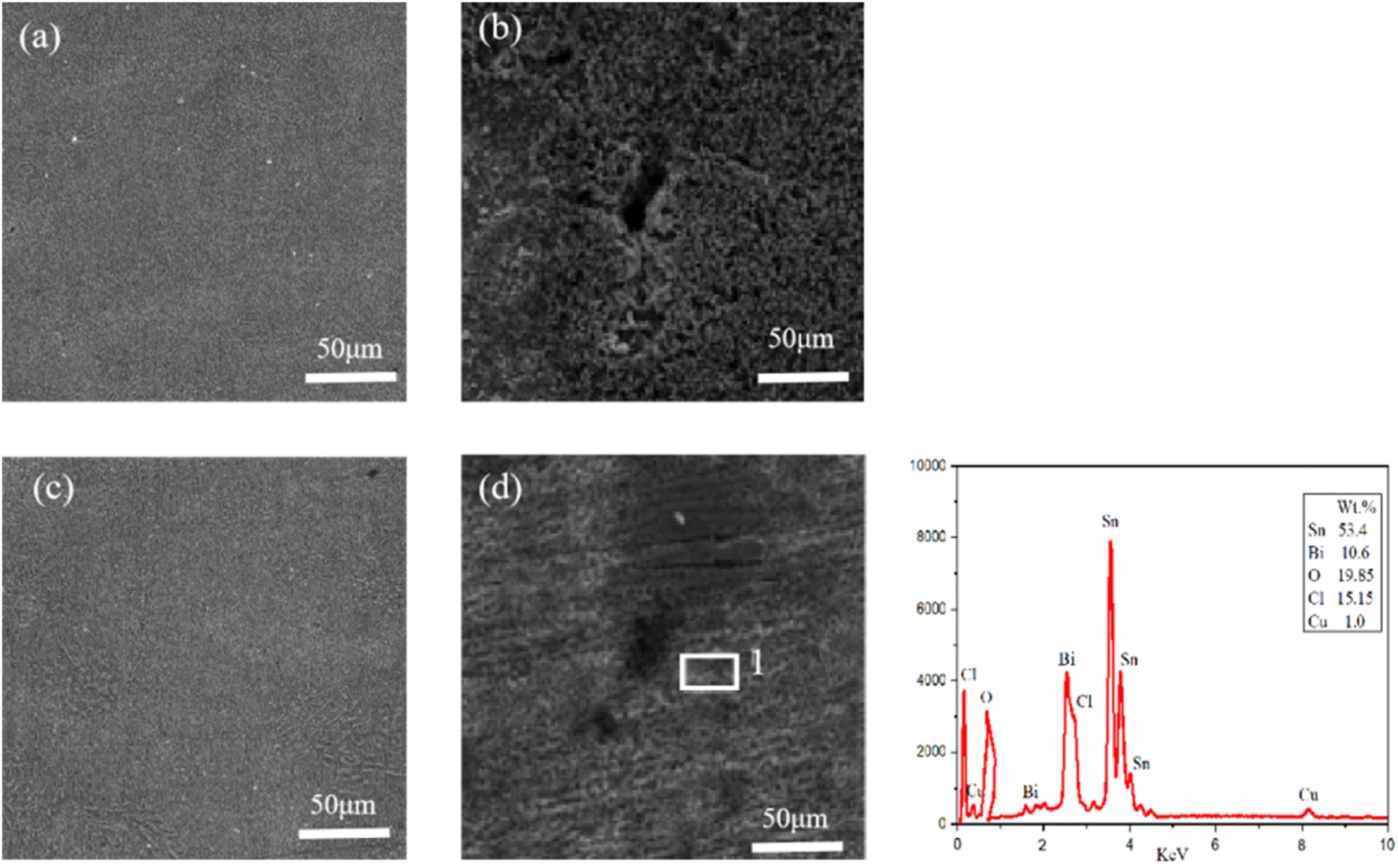
Surface morphology of Sn–0.7Cu–*x*Bi alloy: (a) before corrosion of Sn–0.7Cu alloy; (b) after corrosion of Sn–0.7Cu alloy (c) before corrosion of Sn–0.7Cu–12Bi alloy; (d) after corrosion of Sn–0.7Cu–12Bi alloy.

SEM tests were conducted on the electrochemical corrosion Sn–0.7Cu–*x*Bi alloy, with Sn–0.7Cu alloy and Sn–0.7Cu–12Bi alloy serving as examples. The corrosion morphology, as shown in Fig. 12, revealed that the surface morphology of Sn–0.7Cu alloy was mainly composed of large flake and feather corrosion products. The surface was relatively loose, porous, and uneven in size, with a low stacking density. This indicated that the corroded surface only delayed corrosion, offering weak protection. However, Sn–0.7Cu–12Bi alloy exhibited a surface composed of small, uniform, and dense corrosion products, effectively restraining surface corrosion and providing strong protection. Both alloys have undergone localized corrosion, namely pore corrosion. The Sn–0.7Cu alloy exhibited larger and deeper corrosion pores compared with the Sn–0.7Cu–12Bi alloy. These pores served as channels for corrosive solutions, directly impacting the protective effect of corrosion products. Moreover, larger pores were prone to detachment, resulting in poor corrosion performance. This underscored the superior corrosion resistance of the Sn–0.7Cu–12Bi alloy. The EDS diagram at point 1 of Sn–0.7Cu–12Bi alloy indicated that the surface of the corroded alloy was composed of five elements: Sn, Bi, O, Cl, and Cu. The relatively high percentage of Sn was attributable to its significant proportion in the alloy. Despite the dissolution of Sn at the anode during electrochemical corrosion, a large portion of Sn remained uncorroded. Additionally, combining literature and XRD results, it was concluded that the corrosion products were composed of three compounds: SnO, SnO_2_, and Sn_3_O(OH)_2_Cl_2_, with the flake and feather-shaped corrosion products resulting from the corrosion of Sn.^31^

## 4. Conclusion

This study investigated the impact of Bi addition on the microstructure, mechanical properties, melting properties, and corrosion resistance of Sn–0.7Cu alloy. The conclusions drawn from the study are as follows:

(1) The addition of an appropriate amount of Bi to Sn–0.7Cu alloy improved its overall properties due to the second-phase strengthening and dispersion strengthening. However, an excess of Bi led to supersaturation, coarsened segregation, and reduced alloy properties. The Sn–0.7Cu–*x*Bi alloy was mainly composed of an Sn-rich phase, a Bi phase, and a small quantity of Cu_6_Sn_5_ intermetallic compounds.

(2) The addition of Bi to Sn–0.7Cu alloy played a significant role in grain refinement and dispersion strengthening, increasing the yield strength and tensile strength of the alloy. The tensile strength ranged from 42.97 MPa in Sn–0.7Cu alloy to 156.24 MPa in Sn–0.7Cu–12Bi alloy. Nevertheless, Sn–0.7Cu–15Bi alloy decreased due to the segregation and coarsening of supersaturation Bi in the alloy. The hardness of Sn–0.7Cu–12Bi alloy reached its maximum value at 35.05 HV. However, the addition of Bi significantly reduced the fracture elongation of the alloy and deteriorated its plasticity, with increasing Bi content. The fracture elongation decreased from 37.61% to 11.74%.

(3) The increased Bi content in the alloy led to a continuous decrease in the melting point of Sn–0.7Cu–*x*Bi alloy, accompanied by an expansion of the melting range.

(4) Electrochemical corrosion testing revealed that Sn–0.7Cu–12Bi alloy exhibited superior corrosion resistance with the highest OCP, leading to a self-corrosion potential of −0.92 V. They exhibited the lowest self-corrosion current density of 5.13 × 10^−6^ and the highest *R*_ct_ value of 5.08 × 10^5^. Conversely, pure Sn–0.7Cu alloy displayed the poorest corrosion resistance in a 3.5 wt% NaCl solution. Consequently, the addition of Bi significantly enhanced the corrosion resistance of Sn–0.7Cu alloy. Furthermore, XRD and EDS analyses identified three main corrosion products in the composite alloy: SnO, SnO_2_, and Sn_3_O(OH)_2_Cl_2_.

(5) In summary, Sn–0.7Cu–12Bi alloy exhibited excellent comprehensive properties.

## Data availability

All data included in this study are available upon request by contact with the corresponding author.

## Author contributions

Methodology and writing-review and editing, SGZ; validation, writing-original draft, data curation, visualization, YZ; conceptualization, JHD; Resources and investigation, AYY; supervision and project administration, YC.

## Conflicts of interest

The authors declare that they have no known competing financial interests or personal relationships that could have appeared to influence the work reported in this paper.

## Supplementary Material
